# Supplementary material for: Enhanced Stability and Improved Oral Absorption of Enzalutamide with Self-Nanoemulsifying Drug Delivery System
Source: Int J Mol Sci. 2024 Jan 18;25(2):1197. doi: 10.3390/ijms25021197 (PMC10815963; doi:10.3390/ijms25021197)
Supplement: Supplementary file 1 [file ijms-25-01197-s001.zip › ijms-2795731-supplementary.pdf]

Supplementary Information

Enhanced Stability and Improved Oral Absorption of Enzalutamide with Self-Nanoemulsifying Drug Delivery System

Su-Min Lee [1], Jeong-Gyun Lee [1], Tae-Han Yun [1], Jung-Hyun Cho [2,*] and Kyeong-Soo Kim [1,*]

[1] Department of Pharmaceutical Engineering, Gyeongsang National University, 33 Dongjin-ro,
Jinju 52725, Republic of Korea; m8121@naver.com (S.-M.L.); leepipi87@naver.com (J.-G.L.); xogks7702@naver.com (T.-H.Y.)

[2] Department of Pharmaceutical Engineering, Dankook University, 119 Dandae-ro, Dongnam-gu,
Cheonan 31116, Republic of Korea

* Correspondence: jhcho7301@dankook.ac.kr (J.-H.C.); soyoyu79@gnu.ac.kr (K.-S.K.)

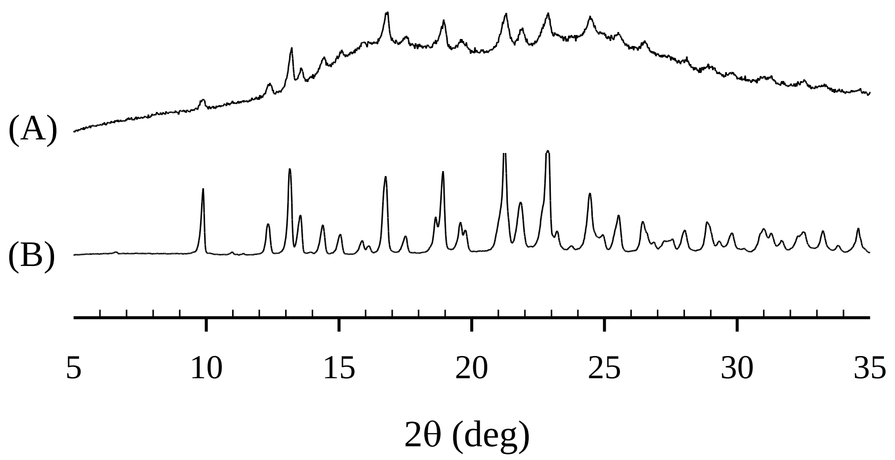

Figure. S1. Powder X-ray diffraction of ENZ8 recrystallized after dispersion in aqueous solution (A) and ENZ crystallin powder (B).

Table S1. The pH solubility of ENZ in various pH (Mean ± S.D.; n = 3).

Test Solution	Solubility (μg/mL)
pH 1.2 buffer	2.08 ± 0.13
pH 4.0 buffer	2.26 ± 0.14
pH 6.8 buffer	1.71 ± 0.04
water	2.01 ± 0.12
